# Antibacterial susceptibility of staphylococcus aureus, salmonella typhi, bacillus subtilis and escherichia coli to snail slime

**DOI:** 10.4314/ahs.v23i4.20

**Published:** 2023-12

**Authors:** Dorcas E Agada, Terdzungwe T Sar, John Adole Ujoh, Linus O Ameh

**Affiliations:** 1 Department of Microbiology, Joseph Sarwuan Tarka University, Makurdi, Benue State, Nigeria; 2 Department of Microbiology, Federal University of Health Sciences Otukpo, Benue State, Nigeria; 3 Department of Biochemistry and Molecular Biology Federal University Dutsinma Kastina State, Nigeria

**Keywords:** Antibacterial, Inhibition, Resistant, Snail, Slime

## Abstract

**Background:**

The emanation of multi-drugs resistant microorganisms and the challenges faced in combating multi-drug resistant infections is a public health issue and this has increased the search for effective antibiotics from natural sources.

**Objectives:**

This work aims to determine the susceptibility of some pathogenic bacterial species to snail slime.

**Methods:**

The antibacterial activity of aqueous and ethanolic snail slime extracts were investigated against *Staphylococcus aureus, Salmonella typhi, Bacillus subtilis* and *Escherichia coli* using the agar well diffusion method.

**Results:**

The results showed that all the organisms were sensitive to both extracts but were more susceptible to aqueous extracts; the highest zone of inhibition for aqueous extracts was 27.33mm ± 2.51mm for *Staphylococcus aureus* at concentration of 1000µl/ml, while the lowest was 11.33mm ± 1.53mm against *Escherichia coli*. The highest zone of inhibition for ethanolic fraction was 15.67 ± 1.15mm for Salmonella typhi. The lowest inhibition was 9.33mm ± 0.58mm for *Escherichia coli*. The MIC was 3.125% for *Staphylococcus aureus, Bacillus subtilis* and *Escherichia coli* and 6.25% for *S. typhi*. The extracts were not cidal at the concentrations used. Statistical analysis revealed that the treatments between the aqueous and ethanolic extracts against *Staphylococcus aureus, Escherichia coli* and *Salmonella typhi* were significant (p ≤ 0.05). The treatment against *B. subtilis* showed no significant difference between the two extracts (p > 0.05).

**Conclusion:**

This study has revealed that snail slime possesses antibacterial properties which can be used as anti-microbial agents against infectious diseases.

## Introduction

The emanation of multi-drug resistant microorganisms has progressively become a global public health issue 1. Pathogens that have developed new resistance mechanisms, resulting to antimicrobial resistance, continue to threaten our ability to combat common infections 2. Cogent and correct use of antibiotics as well as precise infection control measurements is recommended in order to reduce the prevalence of antibiotic resistant bacteria 1. The difficulty in combating multi-drug resistant infections has resulted to an increase in the search for new and effective antibiotics, especially substances originating from natural products.

Snails are mollusks, classified as follows: Kingdom: Animalia, Phylum: Mollusca, Class: Gastropoda, Order: Stylommatophora, Family: Helicidae, Genus: *Helix*, Specie: *aspersa* 3. Morphologically, snails are characterized with a spiral shaped shell which is wound around a spindle. Snails secrete a viscous-elastic substance called Slime with adherent and lubricant properties that also allow them to stick tenaciously to various surfaces. The slime has other importance which include; preventing the snail from dehydration and making it unattractive to potential predators as a result of the slimy nature 4.

Naturally, animals possess their own protective responses against pathogens and their predators. Marine mollusks are exposed to pathogens in their environment, which can be as numerous as 106 bacteria per ml of sea water 5. These protective responses are as well applied to terrestrial mollusks such as the land snail. In order to be defensive against such conditions, mollusks have developed very effective and tactical mechanisms as part of their inherent immunity. These responses include; Secretion of slime and possession of shell by snails 6.

In addition to the benefits to the snail, snail slime has been reported to possess the ability to initiate and speed up wound healing as well as to prevent wound infections on human skin 4. Most bacterial strains have developed resistance to one or multiple drugs 1,2 Since snails secrete slime which helps protect the snail from bacterial attack, this work aims to determine the susceptibility of certain bacterial species to naturally occurring snail slime.

The objectives of this study were to extract snail slime and evaluate the antimicrobial activity of snail slime against some pathogenic bacteria *Staphylococcus aureus, Salmonella typhi, Escherichia coli* and *Bacillus subtilis*.

## Materials and methods

### Study area

Snail species were collected from North Bank in Makurdi town, Benue State, Nigeria. Makurdi is the capital of Benue State, Nigeria 7.

### Collection of snails

Hundred (100) snails were randomly collected from North Bank in Makurdi and identified as *Achatina fulica Ferussac* species by a specialist in the Department of Zoology, Joseph Sarwuan Tarka University, Makurdi. *Achatina fulica Ferussac*, possesses a narrow, conical shell, which is observed to be twice as long as it is wide, containing 7 – 9 whorls when fully grown 8.

### Collection of isolates

Clinically identified isolates were obtained from Benue State University Teaching Hospital Makurdi. Cultures of *Escherichia coli, Staphylococcus aureus, Salmonella typhi* and *Bacillus subtilis* were identified through Gram Staining procedure and biochemical testing (Urease, Catalase, Citrate and Indole) as described by 9.

### Preparation of snail slime extracts

The plastic cage was cleaned prior to the commencement of the experiment; the snails were kept in this cage, fed daily with varieties of leaves and fruits for four weeks. The snails were washed thoroughly using distilled water in order to remove dirt and contaminants from the snail. Cotton wool, dipped in 70% ethanol, was used to disinfect the snail shells before extraction of slime.

Each snail was placed in 15ml of distilled water at room temperature and gently agitated by hand to promote secretion of their slime without being killed in the process. Two fractions of the slime were obtained; the Water-Soluble Fraction (WSF) and the Mucin Fraction (MF) they were obtained by the procedure as described by 10.

The WSF of the slime were filtered to eliminate microbial contamination using sterile ashless filter paper produced by Whatman International Ltd, Maidstone England. Ashless filter paper is 125mm in diameter with pore size 2 – 3 micrometer and Whatman equivalent of No. 42, (Cat No. 1442 125).

### Preparation of bacterial suspensions

Each bacterial isolate was inoculated in sterile physiological saline. The suspensions were adjusted to correspond to 0.5 McFarland Standard, containing 108 CFU/ml of each organism 11.

### Antimicrobial activities (inhibition) Disc diffusion methods

The antimicrobial activities were carried out, observed and measured in alignment with the description by 12.

### Determination of the Minimum Inhibitory Concentration (MIC) and Minimum Bactericidal Concentration (MBC)

The Minimum Inhibitory Concentration (MIC) and Minimum Bactericidal Concentration (MBC) were carried by adhering to the procedure as stated by 13.

## Results

Table 1 shows antibacterial activity of aqueous snail slime against selected bacteria. The highest activity was observed against S. aureus (27.33m) at 1000mg/ml while the lowest activity was observed against *E. coli* (11.33mm) at 100mg/ml. Statistically, no concentration of snail slime showed significant activity against the isolates (p > 0.05).

**Table 1:** Antibacterial activity of aqueous snail slime on selected bacteria

Concentration (mg/ml)	*S. aureus*	Zones of Inhibition (mm) Bacterial Isolates		*B. subtilis*	χ^2^	P
		*E. coli*	*S. typhi*			
1000	27.33 ± 2.51	17.00 ± 2.00	24.33 ± 4.62	18.00 ± 1.73	1.95	0.07
500	23.33 ± 5.51	16.67 ± 5.69	18.00 ± 2.65	15.67 ± 1.15	2.07	0.06
250	17.00 ± 1.00	16.00 ± 1.00	21.33 ± 2.08	16.33 ± 0.58	0.43	0.31
125	23.33 ± 2.89	14.67 ± 2.52	15.67 ± 0.58	13.67 ± 0.58	1.03	0.19
100	15.67 ± 1.15	11.33 ± 1.53	19.00 ± 2.00	11.67 ± 1.15	0.91	0.21

Table 2 shows antibacterial activity of ethanolic snail slime against selected bacteria. The highest activity was seen against *S. aureus* (15.67m) at 125mg/ml and S. typhi (15.67) at 125mg/ml while the lowest activity was seen against E. coli (9.33mm) at 1000mg/ml. Statistically, no concentration of snail slime showed significant activity against the isolates (p > 0.05).

**Table 2:** Antibacterial activity of ethanolic snail slime extract on selected bacteria

Concentration (mg/ml)	*S. aureus*	Zones of Inhibition (mm) Bacterial Isolates		*B. subtilis*	χ^2^	P
		*E. coli*	*S. typhi*			
1000	12.00 ± 2.00	9.33 ± 0.58	12.67 ± 0.58	9.67 ± 0.58	1.28	0.14
500	13.67 ± 1.53	11.33 ± 0.58	12.67 ± 1.15	10.33 ± 0.58	0.12	0.35
250	15.33 ± 2.52	12.67 ± 0.58	12.00 ± 1.00	9.67 ± 0.58	0.93	0.21
125	15.67 ± 1.15	11.67 ± 1.53	15.67 ± 1.53	10.67 ± 1.53	1.1	0.17
100	14.33 ± 0.58	14.00 ± 1.00	14.33 ± 1.53	13.33 ± 0.58	0.63	0.27

Table 3 shows antibacterial activity of aqueous and ethanolic snail slime on selected bacterial isolates. Aqueous slime had greater inhibitory activity on the isolates compared to ethanolic slime, with the highest activity seen on *S. aureus* (23.13mm) while the lowest activity was seen on *E. coli* (11.80mm). Statistically, both aqueous and ethanolic snail slime showed significant activity against *S. aureus, S. typhi* and *E. coli* (p ≤ 0.05); however, both fractions did not show significant activity against *B. subtilis* (p > 0.05).

**Table 3:** Antibacterial activity of aqueous and ethanoic snail slime on selected bacteria

	Zones of Inhibition (mm)			
	Slime Fractions			
Organisms	Aqueous	Ethanolic	χ^2^	P
*S. aureus*	23.13 ± 4.27	14.20 ± 1.46	2.37	0.04
*E. coli*	15.13 ± 2.31	11.80 ± 1.73	2.23	0.05
*S. typhi*	19.40 ± 3.74	13.47 ± 1.50	2.66	0.03
*B. subtilis*	15.07 ± 2.45	10.67 ± 1.53	0.92	0.23

Table 4 shows the antibacterial activity of aqueous and ethanolic snail slime against two Gram Positive (Bacillus subtilis and Staphylococcus aureus) and two Gram Negative (Escherichia coli and Salmonella typhi) bacteria. The inhibitory effect of aqueous slime activity against Gram positive bacteria was observed to be greater when compared to Gram negative bacteria while ethanolic slime was observed to have greater inhibitory activity against Gram negative bacteria compared to Gram positive bacteria. Statistically, there was no significant difference between the activity of these fractions against both Gram positive and Gram-negative bacteria.

**Table 4:** Antibacterial activity of aqueous and ethanolic snail slime against Gram positive and Gram-negative bacteria

	Zones of Inhibition (mm)	
Slime Fraction	Gram Positive	Gram Negative
Aqueous	18.60 ± 4.99	17.27 ± 3.02
Ethanolic	12.44 ± 2.50	12.64 ± 1.18

χ^2^ = 0.04, df = 1, p = 0.32

Table 5 shows the spectrophotometric absorbance of the organisms at various wavelengths. Aqueous fraction was seen to exhibit greater inhibitory activity compared to ethanol fraction, as seen on *S. aureus* (0.61nm) while no inhibitory activity was seen on *S. typhi* (1.20nm). Statistically, there was no significant difference observed between the absorbance values of aqueous and ethanolic fraction (p > 0.05).

**Table 5:** Spectrophotometer absorbance of cultures

	Absorbance (nm)			
Organisms	AF	EF	χ^2^	P
*S. aureus*	0.61 ± 0.22	0.88 ± 0.15	0.07	0.37
*E. coli*	0.67 ± 0.25	0.94 ± 0.11	0.14	0.37
*S. typhi*	0.67 ± 0.25	1.20 ± 0.17	0.23	0.36
*B. subtilis*	0.77 ± 0.15	1.10 ± 0.06	0.14	0.37

KEY: AF = Aqueous Fraction, EF = Ethanolic Fraction

Table 6 shows the Minimum Inhibitory Concentration (MIC) of both aqueous and ethanol snail slime against the bacterial isolates. The MIC (Aqueous) for S. aureus (0.89nm) was at 3.125%, *E. coli* (0.99nm) was at 3.125%, *S. typhi* (380nm) was at 6.250% and *B. subtilis* (350nm) was at 3.125% while the MIC (Ethanolic) for S. aureus (1.11) was at 3.125%, E. coli (600nm) was at 3.125%, B. subtilis (350nm) was at 3.125% but *S. typhi* did not show any inhibition on treatment with ethanol extract.

**Table 6:** Minimum inhibitory concentration of snail slime

FGN	Concentration (%)	
Organism	Aqueous	Ethanolic
*S. aureus* (600nm)	3.125	3.125
*E. coli* (600nm)	3.125	3.125
*S. typhi* (380nm)	6.25	-
*B. subtilis* (350nm)	3.125	3.125

## Discussion

The outcome of the study showed that both the ethanol and aqueous extract of snail slime had inhibitory effect on both Gram positive and Gram-negative test organisms; Bacillus subtilis, Staphylococcus aureus, *Salmonella typhi* and *Escherichia coli* respectively. This observation was previously made by 4 who demonstrated that both aqueous and ethanolic (mucin) fractions showed antibacterial activity against Gram positive bacteria, such as *Bacillus subtilis* and S*taphylococus aureus* as well as Gram negative bacteria such as *Pseudomonas aeruginosa* and *Escherichia coli*. Generally, it was observed that aqueous fraction of the snail slime had greater antibacterial activity against the test organisms than the ethanolic extract. Higher concentrations of aqueous slime showed greater antibacterial activity compared with lower concentrations. Conversely, lower concentrations of ethanolic snail fractions had greater antibacterial activity compared with higher concentrations. This implied that ethanolic treatment reduces the activity of snail slime against the test organisms. However, this was at variance with the work by 14 who demonstrated that ethanolic fraction had more inhibitory effect against bacteria, compared to water-soluble (aqueous) fraction. Ethanol denatures protein 15, the antibacterial components of snail slime are proteins, and this could account for the reason why the ethanolic (absolute) fraction had little effect on the test organisms. 14 used 60% ethanol in extraction, this may account for the ability of the fraction being more effective than aqueous fraction against test organisms.

The aqueous extract was observed to be slightly more potent against Gram positive bacteria, compared to Gram negative bacteria. This observation agrees with the work of 16 who reported that the antimicrobial activity of snail slime was greater against *Staphyloccous aureus*, followed by *Bacillus subtilis*, then by *P. aeruginosa* and finally by *Escherichia coli*.

Achacin, a component of the snail slime is known to inhibit the formation of the peptidoglycan layer and cytoplasmic membrane of bacterial cells 11. Gram positive bacteria are known to possess thick-layer peptidoglycan which is highly prone to the activity of achacin, this may account for the high inhibitory effect of snail slime against Gram positive bacteria compared to Gram negative bacteria. Gram negative bacteria, on the other hand, possess lipopolysaccharide that protects them from harmful substances such as antibiotics.

On measuring the turbidity of the MIC broth using a spectrophotometer, the minimum inhibitory concentration (MIC) result showed that the least concentration/amount of aqueous extract (3.125%) inhibited *Staphyloccocus aureus, Bacillus subtilis* and *Escherichia coli* while *Salmonella typhi* was inhibited at 6.25%.

No ethanolic extract inhibited the growth of *S. typhi* while *Staphyloccocus aureus, Bacillus subtilis* and *Escherichia coli* showed growth inhibition at 3.125%.

The Minimum Bactericidal Concentration of the extracts was recorded to be zero (0) even at the highest concentration used, which was 50%. It was observed that snail slime can only cause bactericidal effect on these organisms at a higher concentration such as 100%.

This agrees with the work of 17 that showed that the slime was able to kill the organisms only at absolute concentrations. This could be as a result of the resistance which microorganisms; especially bacteria are exhibiting against antibiotics.

## Conclusions

This study has revealed that snail slime possesses antibacterial properties which can be used as anti-microbial agents in new drugs for therapy of infectious diseases in humans. Snail slime could be a source of new antibiotic compounds, being less expensive, less toxic to the host microflora than the allopathic drugs.

Although the concentration of snail slime used could not entirely kill the organism, administration of higher doses may be lethal to the organisms.
